# Leitlinienbasierte Aufarbeitung und Befundung von Lymphonodektomiepräparaten und Sentinel-Lymphknoten gynäkologischer Malignome

**DOI:** 10.1007/s00292-020-00805-9

**Published:** 2020-07-22

**Authors:** Anne Kathrin Höhn, Christine E. Brambs, Ramona Erber, Grit Gesine Ruth Hiller, Doris Mayr, Dietmar Schmidt, Elisa Schmoeckel, Lars‑Christian Horn

**Affiliations:** 1 Institut für Pathologie, Arbeitsgruppe Mamma‑, Gynäko- & Perinatalpathologie, Universitätsklinikum Leipzig AöR, Liebigstraße 26, 04103 Leipzig, Deutschland; 2 Frauenklinik des Klinikums rechts der Isar, Technische Universität München, München, Deutschland; 3 Pathologisches Institut, Universitätsklinikum Erlangen, Comprehensive Cancer Center EMN, Friedrich-Alexander-Universität Erlangen-Nürnberg, Erlangen, Deutschland; 4 Pathologisches Institut, Ludwig-Maximilians-Universität München, München, Deutschland; 5 MVZ für Histologie, Zytologie und Molekulare Diagnostik Trier, Trier, Deutschland

**Keywords:** Lymphknoten, Aufarbeitung, Mikrometastasen, Isolierte Tumorzellen, Ultrastaging, Lymph node, Cutting, Reporting, Micrometastases, Isolated tumor cells, Ultrastaging

## Abstract

Die Aufarbeitung von Lymphonodektomiepräparaten gynäkologischer Malignome orientiert sich an den nationalen AWMF-Leitlinien und internationalen Empfehlungen. Die Definition von Mikrometastasen und isolierten Tumorzellen entspricht den Festlegungen der UICC(Union Internationale Contre le Cancer)/TNM(TNM-Klassifikation maligner Tumoren). Deren Nachweis soll im Befundbericht erwähnt werden sowie in die Tumorklassifikation einfließen. Alle übersandten Lymphknoten (LK) sollen untersucht werden mit vollständiger Einbettung aller LK bis 0,3 cm und Lamellierung aller größeren Lymphknoten parallel zu ihrer kurzen Achse in ca. 0,2 cm dicken Scheiben. Bestandteile des histologischen Befundberichtes sind: Zahl der befallenen LK im Verhältnis zur Zahl der entfernten/untersuchten LK entsprechend der Entnahmelokalisationen, metrische Ausdehnung der größten LK-Metastase, Fehlen/Nachweis einer extrakapsulären Ausbreitung. Zuschnitt und Einbettung von Sentinel-LK mit oder ohne Schnellschnittuntersuchung erfolgt in Analogie zu Nicht-Sentinel-LK mit Anfertigung von ca. 3 HE-gefärbten Stufenschnitten in einem Abstand von ca. 200 µm sowohl vom Gefrier- als auch Paraffinblock. Stellen sich die Sentinel-LK in der HE-Färbung negativ dar, soll ein immunhistochemisches Ultrastaging erfolgen.

## Abstract (en)

The handling and reporting of resected lymph nodes in gynecologic cancer follows the recommendations of the German national guidelines and the recommendations of the International Collaboration of Cancer Reporting (ICCR) and the International Society of Gynecologic Pathologists (ISGyP). The definitions of micrometastases and isolated tumor cells are in accordance with the definition of the UICC (Union Internationale Contre le Cancer) and TNM system. Both findings must be reported as part of the pathology report and final tumor classification. It is mandatory to examine all excised lymph nodes with complete processing of all nodes up to 0.3 cm and slicing of all larger nodes in 0.2-cm wide intervals with complete processing of all lamellae. The amount of the resected lymph nodes in correlation to positive nodes, the metric dimension of the largest lymph node metastasis per lymph node region, and the presence of extracapsular extension of the lymph node deposits must be part of the pathology report. The handling and cutting of sentinel lymph nodes are similar to nonsentinel nodes. Within frozen section analyses and final processing from paraffin-embedded sentinel nodes, all nodes should be examined by three-step sections with an interval of about 200 µm. In cases of negative sentinel nodes on H&E staining, immunohistochemical ultrastaging should be performed.

Die Aufarbeitung von Lymphonodektomiepräparaten gynäkologischer Malignome orientiert sich an den jeweiligen AWMF-Leitlinien [3–6] sowie an Empfehlungen der International Collaboration of Cancer Reporting (ICCR), des Royal College of Pathologists und der International Society of Gynecologic Pathologists (ISGyP;[30, 32, 46]).

## Allgemeine Definitionen

Entsprechend der UICC- und TNM-Klassifikation sind *Mikrometastasen* definiert als der histologische Nachweis von Tumorzellen im Lymphknoten von ≥0,2 mm, aber nicht größer als 0,2 cm. Tumorzellen von <0,2 mm Gesamtausdehnung werden als *isolierte Tumorzellen* im Lymphknoten definiert [41, 51].

Ungeachtet der Tatsache, dass die prognostische Bedeutung isolierter Tumorzellen bzw. von Mikrometastasen unklar ist [11, 25, 28, 31, 42, 48], sollen diese, den allgemeinen Empfehlungen der TNM-Klassifikation folgend [51], im Befundbericht enthalten sein und in die Tumorklassifikationen einfließen.

### Infobox 1 Dokumentation von isolierten Tumorzellen und von Mikrometastasen

Der Nachweis von isolierten Tumorzellen bzw. von Mikrometastasen soll im histologischen Befundbericht erwähnt werden und in die TNM-Klassifikation einfließen.

Die *extrakapsuläre Ausbreitung von Lymphknotenmetastasen* ist beim Vulvakarzinom prognose- und stagingrelevant [45, 51]. Beim Zervixkarzinom gibt es Hinweise auf eine prognostische Bedeutung [20]. Beim Vaginal- und Endometriumkarzinom liegen dazu keine Daten vor. Dennoch wird in allen AWMF-Leitlinien empfohlen, die extrakapsuläre Ausbreitung standardmäßig im Befundbericht zu erwähnen [3–6].

*Parametrane (mesometrane) Lymphknoten* gehören beim Endometrium‑, Zervix- und proximalen Vaginalkarzinom zu den regionären Lymphknoten [51] und sollen unter den pelvinen Lymphknoten subsumiert werden. *Intraomentale Lymphknoten* gehören beim Ovarialkarzinom zu den regionären Lymphknoten [22].

## Aufarbeitung und Befundung von Lymphonodektomiepräparaten (Abb. 1 und 2; Tab. 1 und 2)

Das entsprechend der klinisch determinierten Entnahmelokalisation entfernte Fettgewebe soll dreidimensional gemessen werden. Es empfiehlt sich das sorgfältige Lamellieren und Palpieren zur Identifikation aller resezierten Lymphknoten. Sogenannte Clearingtechniken werden in den Leitlinien nicht empfohlen [4–7, 46].

**Figure 1:**
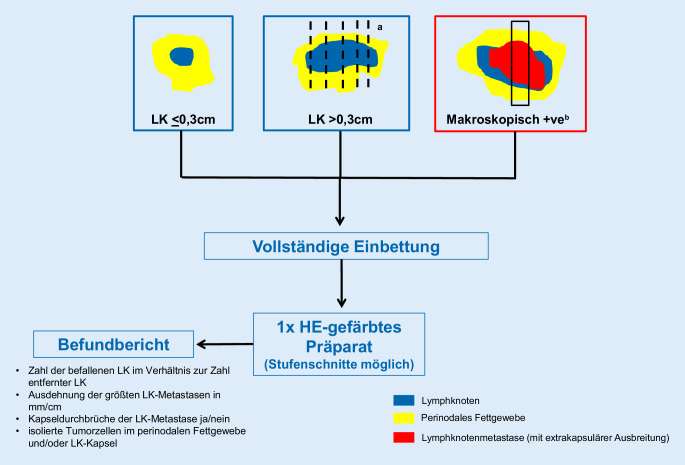

**Figure 2:**
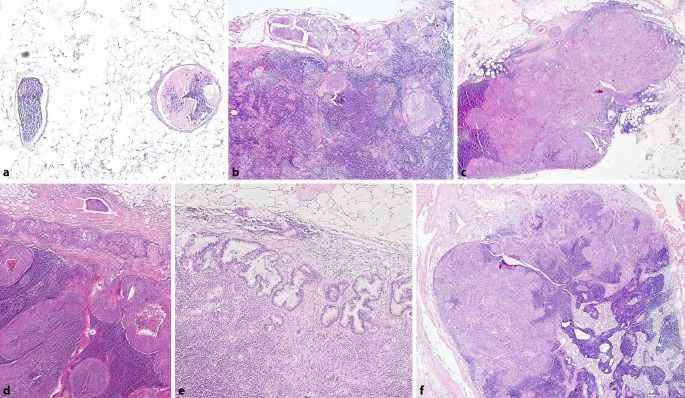

**Table 1:** 

Vulva	Vagina	Zervix	Endometrium	Ovar
– Dreidimensionale Messung des pro Lymphknotenstation resezierten Fettgewebes^a^				
– Lamellieren und Palpieren des resezierten Fettgewebes zur Identifizierung von Lymphknoten				
– Untersuchung ALLER resezierten/identifizierten Lymphknoten				
– Einbettung aller Lymphknoten <0,3 cm in toto				
– Lymphknoten >0,3 cm Lamellierung parallel zur kurzen Achse in 0,2 cm Abständen, komplette Einbettung				
– Belassen eines schmalen perinodalen Fettgewebes zur Beurteilung der Lymphknotenkapsel^b^				
– Bei makroskopisch eindeutig befallenen Lymphknoten Einbettung einer repräsentativen Probe des Lymphknotens				
– Anfertigung eines HE-gefärbten Schnittpräparates pro Block				
– Bei makroskopisch fehlendem Lymphknotennachweis, (ausgedehnte, ggf. komplette) Einbettung des übersandten Fettgewebes^c^				

*HE* Hämatoxylin-Eosin

^a^Einsatz sog. Clearingtechniken nicht empfohlen

^b^Wichtig für die Beurteilung, ob eine extrakapsuläre Ausbreitung der Lymphknotenmetastase(n) vorliegt

^c^Ggf. Einbettung von 2–3 zusätzlichen Kapseln mit Fettgewebe, wenn pro übersandter Lymphknotenstation weniger als 3 Lymphknoten makroskopisch nachweisbar sind

**Table 2:** 

Vulva	Vagina	Zervix	Endometrium	Ovar
– Angabe der Zahl der befallenen Lymphknoten im Verhältnis zur Zahl der entfernten/untersuchten Lymphknoten in Zuordnung zur Entnahmelokalisation (z. B. 4/13 inguinal links oder 0/15 inguinal links bzw. 7/22 iliakal extern rechts oder 0/17 paraaortal)				
– Angabe der größten Ausdehnung der größten Lymphknotenmetastase in mm/cm				
– Angabe des Fehlens/Nachweises eines Kapseldurchbruches der Lymphknotenmetastase(n)				
– Angabe des Nachweises isolierter Tumorzellen im Lymphknoten sowie des Nachweises von Lymphgefäßeinbrüchen im perinodalen Fettgewebe und/oder der Lymphknotenkapsel				

Alle makroskopisch identifizierten Lymphknoten sollen histologisch untersucht werden. Dabei sollten Lymphknoten bis ca. 0,3 cm Größe komplett eingebettet und größere Lymphknoten parallel zu ihrer kurzen Achse in ca. 0,2 cm dicke Scheiben lamelliert und ebenfalls komplett eingebettet werden [13, 18, 27, 30]. Bei makroskopisch befallenen Lymphknoten ist die Einbettung einer repräsentativen Probe ausreichend. Zur Beurteilung einer extrakapsulären Ausbreitung empfiehlt es sich, einen schmalen Fettgewebssaum an den Lymphknoten zu belassen [23, 30]. Lassen sich im übersandten Fettgewebe makroskopisch keine Lymphknoten nachweisen, empfiehlt sich eine ausgedehnte, ggf. komplette Einbettung und Aufarbeitung. Beim Nachweis von <3 Lymphknoten im lokalisationsbezogenen Fettgewebe empfiehlt es sich, 1–3 zusätzliche Kapseln zum Nachweis mikroskopisch kleiner Lymphknoten einzubetten.

### Infobox 2 Aufarbeitung von Lymphonodektomiepräparaten

Bei Lymphonodektomiepräparaten bei gynäkologischen Malignomen sollen alle entfernten Lymphknoten histologisch untersucht werden.

Lymphknoten bis ca. 0,3 cm Größe sollten komplett eingebettet und größere Lymphknoten parallel zu ihrer kurzen Achse in ca. 0,2 cm dicke Scheiben lamelliert und ebenfalls komplett eingebettet werden.

Außerhalb von Studien genügt die Anfertigung eines HE-gefärbten Schnittes pro Block [3–6, 32]. Bei allen gynäkologischen Malignomen haben Studien ergeben, dass die Anfertigung von Stufenschnitten (mit einem Abstand von ca. 200 µm) die Chance der Detektion kleiner Metastasen bzw. von Mikrometastasen sowie isolierter Tumorzellen erhöht [8, 14, 21, 35, 36].

Die metrische Größenausdehnung von Lymphknotenmetastasen ist beim Vulva- und Ovarialkarzinom stagingrelevant [51]. Beim Zervixkarzinom gibt es Hinweise auf die prognostische Bedeutung der Unterscheidung zwischen Mikro- und Makrometastasen [19, 43].

Eine extrakapsuläre Ausbreitung von Lymphknotenmetastasen ist beim Vulvakarzinom bedeutsam für das Staging [51]. Für das Ovarial- und Zervixkarzinom scheint die extrakapsuläre Ausbreitung prognoserelevant zu sein [16, 19, 34].

Anforderungen an den histologischen Befundbericht bei Lymphonodektomiepräparaten sind ([9, 12, 18, 26, 27], Abb. 1; Tab. 2):

- Angabe der Zahl der befallenen Lymphknoten im Verhältnis zur Zahl der entfernten/untersuchten Lymphknoten in Zuordnung zur Entnahmelokalisation (z. B. 4/13 inguinal links oder 0/15 inguinal links bzw. 7/22 iliacal extern rechts oder 0/17 paraaortal),
- Angabe der größten Ausdehnung der größten Lymphknotenmetastase in mm/cm,
- Angabe des Fehlens/Nachweises eines Kapseldurchbruches der Lymphknotenmetastase,
- Angabe des Nachweises isolierter Tumorzellen im Lymphknoten.

## Aufarbeitung und Befundung von Sentinel-Lymphknoten (Abb. 3 und 4; Tab. 3)

### Konventionelle Aufarbeitung

Zur histopathologischen Untersuchung von Sentinel-Lymphknoten gynäkologischer Malignome gibt es derzeit kein einheitliches Protokoll [8, 9, 13, 17, 30]. Konsens besteht dahingehend, dass in jedem Fall ein Ultrastaging erfolgen soll [13, 17, 30]. Der Einsatz der Sentinel-Lymphknotentechnik beim Ovarialkarzinom steht derzeit am Anfang [10].

Aufgrund der Ergebnisse großer Studien sollen der/die klinisch identifizierten Lymphknoten vollständig eingebettet werden. Dabei ist es sinnvoll, diese in ca. 0,2 cm starke Scheiben zu lamellieren und komplett einzubetten [9, 13, 15, 32, 37, 46, 46, 50]. Dabei können mehrere Lamellen in einem Paraffinblock eingebettet werden. Von den Paraffinblöckchen sollen (mindestens) 3 HE-gefärbte Stufenschnitte jeweils in einem Abstand von ca. 200 µm angefertigt werden [9, 13, 14, 23, 26, 29, 32, 35, 36].

#### Infobox 3 Aufarbeitung von Sentinel-Lymphknoten gynäkologischer Malignome

Sentinel-Lymphknoten gynäkologischer Malignome sollen vollständig eingebettet und in Stufenschnitten untersucht werden. Zusätzlich sollen bei in der HE-Färbung karzinomfreien Sentinel-Lymphknoten immunhistochemische Untersuchungen durchgeführt werden (sog. Ultrastaging).

Lassen sich in den HE-gefärbten Schnittpräparaten keine Tumorzellen nachweisen, ist eine immunhistochemische Untersuchung mit einem (oder mehreren) Panzytokeratinantikörper(n) sinnvoll [13, 15, 26, 33, 35, 39, 48, 50]. Für Vulvakarzinome wird dieses Vorgehen bereits von der Leitlinie gefordert.

**Figure 3:**
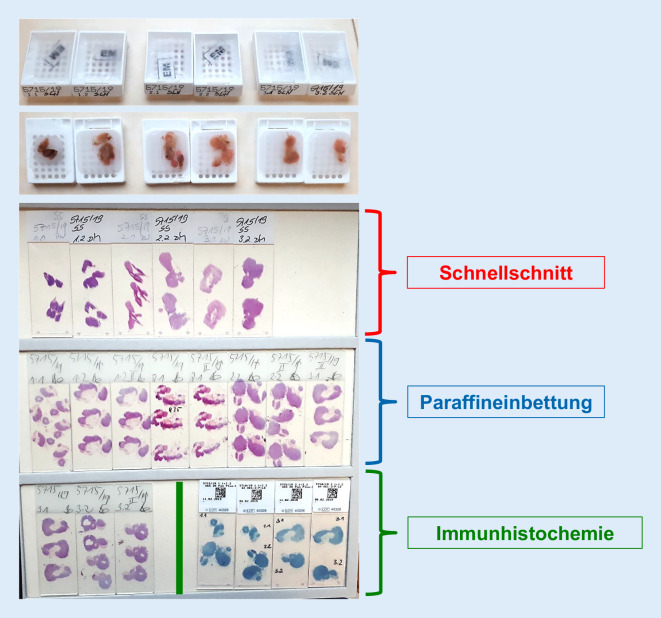

**Figure 4:**
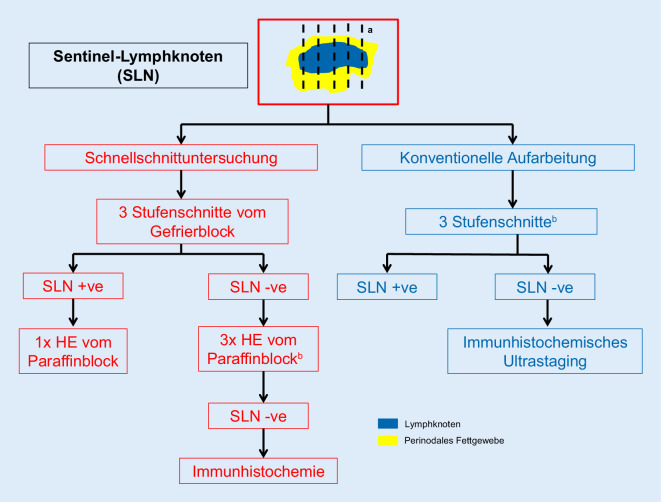

**Table 3:** 

	Vulva	Vagina	Zervix	Endometrium	Ovar
*1) Einbettung*	– Dreidimensionale Messung des resezierten Fettgewebes				
	– Lamellieren und Palpieren des resezierten Fettgewebes zur Identifizierung von Lymphknoten				
	– Untersuchung ALLER resezierten/identifizierten Lymphknoten				
	– Lamellieren in 0,2-cm-Intervallen				
	– Vollständige Einbettung dieser Lamellen (mehrere Lamellen in einem Block möglich)				
*2) Konventionelle Aufarbeitung*	– Anfertigung von (mindestens) 3 HE-gefärbten Stufenschnitten vom Paraffinblock				
	– Intervall zwischen den Schnittstufen ca. 200 µm				
*3) Intraoperative Schnellschnittuntersuchung*	– Anfertigung von (mindestens) 3 HE-gefärbten Stufenschnitten vom Gefrierblock				
	– weitere Aufarbeitung wie unter 2) beschrieben				
*4) Immunhistochemisches Ultrastaging *(von in der HE-Färbung negativen Sentinel-Lymphknoten)	– 1 oder 2 Panzytokeratinantikörper (Zytokeratincocktails)				
	– Ggf. Einsatz organspezifischerer Antikörper wie p16^a^, CK7^b^, HMB-45^c^, Melan A^c^				

*HE* Hämatoxylin-Eosin

^a^Zu beachten ist, dass es p16-negative Karzinome gibt

^b^CK7 kann bei einem Teil der Endometriumkarzinome negativ sein

^c^Einsatz beim malignen Melanom der Vulva, ggf. auch bei vaginalen Schleimhautmelanomen

Die alleinige Verwendung eines Antikörpers gegen p16 beim Vulva‑, Vaginal- und Zervixkarzinom ist nicht sinnvoll, da aufgrund der unterschiedlichen Pathogenesewege nicht alle Karzinome dieser Lokalisationen HPV-positiv sind [1, 2, 38, 40, 44].

Der immunhistochemische Nachweis zytokeratinpositiver Strukturen ohne Kern innerhalb eines Sentinel-Lymphknotens beim Vulvakarzinom scheint keine biologische Relevanz zu besitzen [47], ist jedoch häufig mit einer lymphogenen Metastasierung assoziiert, sodass die entsprechenden Lymphknoten weiter mittels Stufenschnitten bzw. immunhistochemisch aufgearbeitet werden sollten.

Beim malignen Melanom erfolgt die immunhistochemische Untersuchung mit melanomspezifischen Markern [13].

### Schnellschnittuntersuchung

Für die intraoperative Schnellschnittuntersuchung von Sentinel-Lymphknoten bei gynäkologischen Malignomen gibt es keine allgemeingültigen Richtlinien [24, 32, 48–50].

Die ESGO-Leitlinie zum Zervixkarzinom [9, 12] sowie die überarbeitete Version der S3-Leitlinie zum Zervixkarzinom, die auf die anderen Malignome extrapoliert werden kann, empfehlen (Tab. 3):

- Makroskopische Aufarbeitung wie oben beschrieben.
- Untersuchung aller Sentinel-Lymphknoten im Schnellschnitt.
- Bei makroskopisch sichtbarem Tumor ist die intraoperative Untersuchung einer Probe des befallenen Lymphknotens ausreichend.
- Makroskopisch unauffällige Lymphknoten sollen vollständig intraoperativ untersucht werden.
- Von den Gefrierblöckchen sollen (3) Stufenschnitte angefertigt werden.
- Die histologische Gefrierschnittuntersuchung kann durch eine intraoperative Imprintzytologie ergänzt werden.

Untersuchungen zum Sentinel-Lymphknoten bei gynäkologischen Malignomen geben verschiedene Aufarbeitungstechniken, einschließlich der Zahl und Anfertigung von Stufenschnitten an [9, 15, 23, 26, 32, 35, 36, 50]. In Analogie zur Aufarbeitung der Sentinel-Lymphknoten im Paraffinblock erscheint die Anfertigung von 3 Stufenschnitten vom Gefrierblock sinnvoll.

Bei im Schnellschnitt und in der anschließenden Paraffinaufarbeitung tumorfreien (Sentinel‑)Lymphknoten sollen die Aufarbeitung und das Ultrastaging wie oben beschrieben erfolgen.

## Fazit für die Praxis

- Die Definition von Mikrometastasen und isolierten Tumorzellen gynäkologischer Malignome ist von der UICC/TNM festgelegt.
- Der Nachweis von Mikrometastasen und isolierten Tumorzellen soll im Befundbericht erwähnt werden und in die TNM-Klassifikation einfließen.
- Alle übersandten Lymphknoten (LK) sollen untersucht werden mit vollständiger Einbettung aller LK bis 0,3 cm und Lamellierung aller größeren LK parallel zu ihrer kurzen Achse in ca. 0,2 cm dicken Scheiben.
- Bestandteile des histologischen Befundberichtes sind: Zahl der befallenen LK im Verhältnis zur Zahl der entfernten/untersuchten LK, metrische Ausdehnung der größten LK-Metastase, Fehlen/Nachweis einer extrakapsulären Ausdehnung.
- Zuschnitt und Einbettung aller Sentinel-LK wie beschrieben mit Anfertigung von (mindestens) 3 Stufenschnitten in einem Abstand von ca. 200 µm.
- Bei in der HE-Färbung negativen Sentinel-LK erfolgt ein immunhistochemisches Ultrastaging.
- Schnellschnittuntersuchung von Sentinel-LK: Zuschnitt und Einbettung wie beschrieben, Anfertigung von 3 Stufenschnitten vom Gefrierblock.
